# The Effect of Leptin and Adiponectin on KiSS-1 and KissR mRNA Expression in Rat Islets of Langerhans and CRI-D2 Cell Line

**DOI:** 10.5812/ijem.15297

**Published:** 2014-04-01

**Authors:** Mandana Mahmoodzadeh Sagheb, Negar Azarpira, Ramin Yaghobi

**Affiliations:** 1 Department of Biology, Kazeroon Branch, Islamic Azad University, Kazeroon, IR Iran; 2 Transplant Research Center, Shiraz University of Medical Sciences, Shiraz, IR Iran

**Keywords:** Leptin, Adiponectin, Kisspeptins, Kiss1r Protein, Mouse, Islets of Langerhans

## Abstract

**Background:**

Leptin and adiponectin are the two key metabolic hormones secreted from adipocytes to control food intake and energy expenditure. The action of both hormones in regulation of Gonadotropin Releasing Hormone (GnRH) secretion from the hypothalamus is mediated through Kisspeptins. Kisspeptins are products of KiSS-1 gene. Leptin and adiponectin are modulators of KiSS-1 expression in the hypothalamus. These peptides have also important roles in pancreatic β-cells to control insulin synthesis and secretion and their receptors are detected in Langerhans islets. We hypothesized that leptin and adiponectin might alter KiSS-1 and Kiss Receptor mRNA expression in the islets.

**Objectives:**

The aim of this study is to investigate any modulatory effect that leptin and adiponectin may have on the expression of Kiss-1 and KiSSR gene in Langerhans islets.

**Materials and Methods:**

We isolated the islets from adult male rats by collagenase and cultured CRI-D2 cell lines to investigate the effect of leptin and adiponectin. Then, we incubated them with different concentrations of leptin and adiponectin for 24 hours. After that, RNA was extracted from the islets and CRI-D2 cells and transcripted to cDNA. KiSS-1 and KissR expression levels were evaluated by real time PCR.

**Results:**

In islet and CRI-D2 cells, leptin increased the KiSS-1 mRNA expression significantly, but adiponectin decreased it was expected.

**Conclusions:**

These findings indicated the possibility that KiSS-1 mRNA expression is a mediator of leptin and adiponectin function in the islets.

## 1. Background

Leptin and adiponectin are adipokines which regulate the insulin sensitivity and energy homeostasis (1). Leptin decreases the insulin sensitivity, while adiponectin increases it (2). The plasma level of leptin is proportional to the body fat content and Body Mass Index (BMI) (3, 4). On the other hand, unlike leptin, adiponectin systemic concentration is negatively related to adiposity (5). 

Leptin has been known as a key regulator of food intake and energy expenditure. This hormone transmits the signals of satiety and body fat stores to the brain, especially hypothalamus (6, 7). Recent studies have also shown the existence of adiponectin receptors Adipo1 and Adipo2 in the hypothalamus and the involvement of adiponectin in the central regulation of appetite and energy homeostasis in rodents and humans (8).

Moreover, leptin seems to transmit the information of metabolic status and energy stores of the body to the hypothalamus to control reproduction. Uncontrolled diabetes is associated with reproductive abnormalities and hypogonadotropic hypogonadism is frequently observed in models of experimental diabetes (9). However, expression of leptin receptor in GnRH neurons is little or zero (10); of course, there are some Kiss neurons in the hypothalamus, which express KiSS-1 mRNA. The KiSS-1 gene encodes 54, 14, 13, and 10 amino-acid peptides, known as kisspeptins (11). Kisspeptins have been detected in various tissues, such as placenta, pancreas, testes, and central nervous system, and are known as a “molecular switch for puberty”. About 75% of GnRH neurons coexpress KiSS-1 (12). These hypothalamic Kiss-1 neurons also express leptin receptor and have been suggested to modulate the reproductive function by increasing or decreasing GnRH secretion (9). Hypothalamic KiSS-1 mRNA expression is significantly reduced in ob/ob compared to wild-type mice, while increased in ob/ob (leptin deficient) mice treated with leptin (13). Moreover, administration of leptin increases hypothalamic KiSS-1 mRNA as well as LH and testosterone concentrations in hypogonadotropic diabetic male rats (13). Convincing evidence proposes that leptin is able to modulate Kiss-1 expression in the hypothalamus (9). 

The effects of leptin are not restricted to the hypothalamus. Leptin receptor mRNA was also detected in rat islets and pancreatic β-cell line (7, 14). The inhibitory effect of leptin on insulin secretion of pancreatic β-cells has been established in the previous studies (7, 15). Since pancreatic beta cells express KiSS-1 and kiss receptor (12), we hypothesized that the modulatory impact of leptin might be through Kiss-1 mRNA expression. To test this hypothesis, we evaluated KiSS-1 transcription in rat islets of Langerhans and CRI-D2 beta cell line after leptin treatment.

Moreover, Adiponectin has a role in controlling the reproductive system. Recently, the expression of adiponectin and its receptors has been confirmed in rat ovary and testis (16). Moreover, adiponectin considerably inhibited GnRH secretion from GT1-7 hypothalamic GnRH neuron cells (17). This hormone reduced the release of LH from rat pituitary cells, as well (18). Recently, it has been shown that adiponectin inhibits KiSS-1 gene transcription in the hypothalamic GT1-7 neurons (19).

Adiponectin receptors (AdipoR1/2) have been identified in the pancreatic islet cells (20); however, functional effect of adiponectin and involvement of other molecules in adiponectin induced signaling are not completely understood.

## 2. Objectives

The main aim of the present study was to investigate any modulatory effect that leptin and adiponectin may have on the expression of KiSS-1 and KissR may have on Langerhans islets.

## 3. Materials and Methods

### 3.1. Islets Isolation

In this study, adult Wistar male rats weighing 300-350 grams were maintained at 12 h light, 12 h dark condition at 22- 24°C temperature with free access to pelleted food and tap water. 

All the animal experiments were approved by the Departmental Committee for Care and Use of Laboratory Animals.

Rats were anesthetized with ketamine (100 mg/kg ip) and islets of Langerhans were isolated by collagenase P (Roche, Germany). Besides, pancreatic duct was cannulated with PE50 tube (Becton Dickinson Company) and collagenase P (15 mg in 15mL HBSS (Hank’s Balance Salt Solution)) was injected to the duct. The distended pancreas was maintained at 37ºC for 25 min. Then, the islets were washed by medium A (HBSS, 1% HEPES (Sigma-Aldrich, USA), 2%FBS) for 5 times and by means of Lymphoprep (Axis-Shield, Norway), the islets were isolated and purified from exocrine cells. Islet size and purity were determined by microscopic sizing on a grid after staining with 1, 5-diphenylthiocarbazone (DTZ). Afterwards, the islets were incubated in 5% CO_2_ in RPMI 1640 (Sigma-Aldrich, USA) supplemented with 10% fetal bovine serum and 1% penicillin-streptomycin antibiotic (Invitrogen) at 37ºC for 24 h before use.

### 3.2. Cell Culture

We used rat islets of Langerhans to investigate whether the transcription of KiSS-1 and KissR genes could be directly regulated by leptin and adiponectin. The experiment was also repeated with CRI-D2 cells to confirm the results.

CRI-D2 cell lines were supplied by national cell bank of Iran (NCBI), Pasteur Institute (Tehran, Iran) and the detailed profile is provided at http://hpacultures.org.uk. The islets were seeded into 48-well plates at 100 islets per well and CRI-D2 cell line was placed in 6-well plates at 105 cells per well and incubated at 37^o^C in an atmosphere of 5% CO_2_ in RPMI supplemented with 10% fetal bovine serum and 1% penicillin-streptomycin for 24 hours before use.

### 3.3. Islets Treatment With Leptin and Adiponectin

After 24 hours of incubation, the medium was replaced with medium alone or containing 3.125, 6.25, 12.5, 25, and 50 nmol/L leptin (Sigma-Aldrich, USA) or 2.5, 5, and 10 µg/mL adiponectin in each well and then incubated for 24 hours. 

### 3.4. RNA Isolation and cDNA Synthesis

Total RNA was extracted from the islets and cell line by RNA kit II (Invitek, Germany) according to the manufacturer’s instructions. The extracted RNA was quantitated by OD260/280 measurement. Extracted RNA (10 μg) was reverse transcribed in a final volume of 20-μL with random hexamer primers, using cDNA First Strand Synthesis kit (Fermentas, Life Science, EU).

### 3.5. Quantitative Real Time PCR

At first, 5 µg of cDNA was added to taq man master mix (Takara, Takara Shuzo., Otsu, Japan). The final volume of the PCR was 20 μL: 10 μL Master Mix, 0.6 μL of each primer, 0.6 μL probe, 0.4 μL reference dye, and 2.8 μL dH_2_O. PCR amplification was performed by Step One Plus (ABI prism 7500, step one plus) real time PCR detection system under the following conditions: 10 minutes at 95ºC and 10 seconds at 95ºC and 30 seconds at 60ºC for 40 cycles. The primers and probes for real-time PCR were designed using rat genomic sequences as templates through NCBI (http://www.ncbi.nlm.nih.gov/pubmed) and Allele ID programs. Furthermore, GAPDH was selected as the endogenous control and the transcription of Kisspeptin and Kiss receptor was checked relative to GAPDH by using the Relative quantification method as follows:

∆Ct = (Ct target gene, treatment- Ct GAPDH, treatment)- (Ct target gene, control - Ct GAPDH, control)

RQ = 2^-∆∆Ct^

Each experiment was repeated 3 times. The sequences of the primers and probes are listed in Table 1.

**Table 1.** The Sequence of Primers and Probes

	Forward Primer	Reverse Primer	Probe
**GAPDH**	5'GGCTCTCTGCTCCTCCCTGTTC3'	5'CGGCCAAATCCGTTCACACCGA3'	5'GCCGCATCTTCTTGTGCAGTGCCAGCC3'
**KiSS-1**	5'ATGATCTCGCTGGCTTCTTGG3'	5'GGTTCAGGGTTCACCACAGG3'	5'TGCTGCTTCTCCTCTGTGTGGCCT3'
**kissR**	5'TTTCCTTCTGTGCTGCGTACC3'	5'CGAGACCTGCTGGATGTAGTTG3'	5'CGCTCCTCTATCCGCTGCCCACCT3'

### 3.6. Statistical Analysis

The data are shown as mean ± SD. For evaluation of each gene transcription, 3-5 test groups were compared to the controls and each experiment was repeated 3 times. Different groups were compared through one-way ANOVA followed by Tukey’s test for pair wise comparison. P value < 0.05 was considered as statistically significant. The statistical analyses and design of the graphs were performed using the Graph Pad Prism 5 software, by California corporation).

## 4. Results

### 4.1. The Effect of Leptin on KiSS-1 and KissR mRNA Level in Rat Islets of Langerhans and CRI-D2 Cell Line

**Figure 1.**
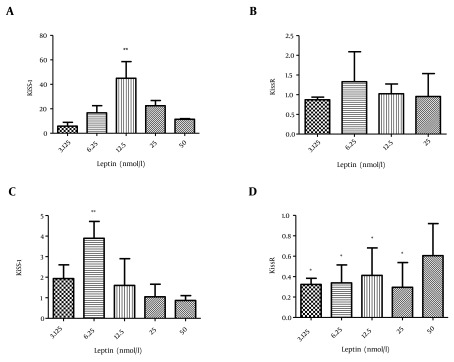
The Effect of Different Concentrations of leptin Treatment (3.125, 6.25, 12.5, 25 and 50 nmol/L) for 24 Hours on Gene Transcription a) The effect of leptin on KiSS-1 transcription in rat islets of Langerhans, b) Leptin effect on KissR transcription in rat islets of Langerhans, c) Effect of leptin on KiSS-1 transcription in CRI-D2 cells, d) Effect of leptin on KissR transcription in CRI-D2 cells. Results are shown as relative fold change in the Kiss-1 and KissR expression with respect to the control (untreated). * P < 0.05. ** P < 0.01

To determine the effect of leptin, first we investigated its effect on rat islets of Langerhans. Leptin (12.5 nmol/L) significantly elevated the transcription of Kiss-1 compared to the control group (P value < 0.01); however, no considerable change was observed in KissR transcription (Figure 1 a, b). 

We also studied the effect of leptin treatment on CRI-D2 cells on the transcription of KiSS-1 and KissR genes. According to the results, 6.25 nmol/L leptin increased the transcription of kiss-1 in comparison to the control group (P value < 0.05). In addition, the transcription of KissR was considerably reduced after the incubation of CRI-D2 cells with 3.25, 6.25, 12.5, and 25 nmol/L leptin (P value < 0.05) (Figure 1 c, d).

### 4.2. The Effect of Adiponectin on KiSS-1 and KissR mRNA Expression in Rat Islets of Langerhans and CRI-D2 Cells

**Figure 2.**
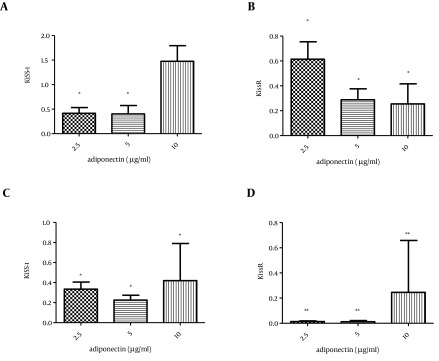
The Effect of Different Concentrations of Adiponectin Treatment (2.5, 5 and 10 µg/mL) for 24 Hours on KiSS-1 and KissR Gene Transcription A) The effect of adiponectin on KiSS-1 transcription in rat islets of Langerhans, B) Adiponectin effect on KissR transcription in rat islets of Langerhans, C) Effect of adiponectin on KiSS-1 transcription in CRI-D2 cells, D) Effect of adiponectin on KissR transcription in CRI-D2. Results are shown as relative fold change in the Kiss-1 and KissR expression with respect to the control (untreated). * P < 0.05. ** P < 0.01

Low doses of adiponectin (2.5, 5 µg/mL) decreased the KiSS-1 transcription significantly (P-value < 0.05), while its higher dose (10 µg/mL) did not alter KiSS-1 mRNA level in the islet cells. On the other hand, the transcription of KissR significantly decreased in all the concentrations of adiponectin (Figure 2 e, f).

Furthermore, treatment of CRI-D2 cells with adiponectin significantly decreased both KiSS-1 (P value < 0.05) and KissR (P value < 0.01) transcription (Figure 2 g, h).

## 5. Discussion

In this study, we investigated the effect of leptin on gene expression in islets of Langerhans. It has been reported that leptin inhibits insulin biosynthesis and secretion in pancreatic β-cells (21). In addition, the expression of long isoform of leptin receptor (ObRb) in rat islet cells was demonstrated and it proves the function of leptin in the pancreatic islets (7). Recently, involvement of leptin in regulation of Kiss-1 and KissR expression in the hypothalamus was elucidated (22). According to these documents, we proposed the possibility that leptin effect on the pancreatic islets might be mediated by KiSS-1 and its receptor mRNA expression. Therefore, we investigated the role of leptin on Kiss-1 and KissR mRNA expression in rat islets of Langerhans and CRI-D2 cell line. In the present study, we showed that leptin increased Kiss-1 mRNA expression in the islets and CRI-D2 cells. Previously, it was shown that KiSS-1 expression in leptin-deficient ob/ob mice was lower than the normal mice (23) and central leptin infusion corrected reduced KiSS-1 expression observed in streptozotocin-induced diabetic male rats. Moreover, KiSS-1 and KissR expression in cultured human fetal GnRH-secreting neuroblasts was increased by adding leptin (24). Additionally, leptin stimulated KiSS-1 mRNA expression in the mouse hypothalamic cell line N6 (25). In this experiment, leptin decreased KissR mRNA in CRI-D2 cell line, which might have resulted from a negative feedback.

Current studies have also illustrated the involvement of adiponectin in altering energy expenditure (26) and reproductive function (27). It has also been demonstrated that adiponectin inhibited GnRH secretion from GT1-7 hypothalamic GnRH secreting neurons. Recently, the effect of adiponectin on hypothalamic KiSS-1 gene transcription, which is the upstream signal of GnRH, has been studied (19). These findings showed that similar to leptin, adiponectin has also a modulatory effect on KiSS-1 expression in GT1-7 neurons in the hypothalamus (19). In addition to its important central roles, adiponectin is functionally active in rodents (20) and humans islets of Langerhans (28). Further evidence for a physiological link between adiponectin and glucose homeostasis resulted from some observations which showed that adiponectin increased insulin secretion (29) and regulated beta-cell viability as well as gene expression (30). Although KiSS-1 and its receptor GPR54 were identified in the pancreatic islets before, the physiological factors affecting its transcription are not fully understood. In this study, we investigated the role of adiponectin in transcriptional control of KiSS-1 and KissR in islets and CRI-D2 cells. To the best of our knowledge, the current study is the first one, which showed that adiponectin inhibited the transcription of these two genes. Therefore, those opposite effects of leptin and adiponectin observed in insulin secretion and glucose homeostasis were found in this study as well. Of course, further confirmation of these primary results need the application of western blotting assay. The study findings for the first time characterized the functional relevance of putative key mediators, leptin and adiponectin, on KiSS-1 and KissR mRNA expression in the rat islets of Langerhans and CRI-D2 cells. Therefore, these genes expression might be the target for new treatment strategies for metabolic disorders like obesity and diabetes mellitus.
